# Socio-demographic determinants of body mass index among school children in Ebonyi State, Nigeria

**DOI:** 10.4102/phcfm.v10i1.1450

**Published:** 2018-03-07

**Authors:** Henry A. Akinsola, Chinwe Ezeruigbo, Kwabena A. Kyei, Felix C. Anyanwu, Robert Nemakhavhani

**Affiliations:** 1 Department of Public Health, University of Venda, South Africa; 2 Department of Nursing, Ebonyi State University, Nigeria; 3 Department of Statistics, University of Venda, South Africa; 4 Department of Community Services, Vhembe District Municipality, South Africa

## Abstract

**Background:**

African specific studies on the factors associated with the growth pattern of children are needed to guide evidence and develop effective population-based interventions that can be tailored to the unique African context.

**Purpose:**

The purpose of this study was to determine the socio-economic correlates of body mass index (BMI) of primary and secondary school children in Ebonyi State, which is situated in south-eastern Nigeria.

**Methodology:**

This was a quantitative, cross-sectional study that utilised clustering and stratified sampling techniques to select 1000 learners from primary and secondary schools located in Abakaliki local government area. Apart from a questionnaire, a Mettler weighing scale was also used for data collection. A generalised linear model was used to test the association between the participants’ socio-demographic characteristics and their BMI.

**Result:**

BMI was positively related to female gender but negatively related to age and level of education. The mode of cooking, who the participants live with and how they get to school also predicted changes in BMI. Other variables like mother’s occupation and family’s mode of transport were also associated with BMI changes while father’s level of education, mother’s level of education, father’s occupation and the type of residence did not have any statistical relationship with BMI.

**Conclusion:**

The present study shows that the BMI of school children is influenced by the socio-demographic characteristics surrounding them. Therefore, efforts should be made to improve the socio-economic standing of families in this community.

## Introduction

In different parts of the world, especially in developed countries, studies have shown that both the economic and social context of a child, including a school-age child, are associated with the body mass index (BMI).^1,2,3,4,5,6,7,8,9^ McKay et al.^1^ conducted a survey to examine the influence of economic and social context in relation to abnormal weight and the result showed that both social and economic indicators were predictors of having an above-normal BMI. In a study conducted in the United States,^2^ children from lower-income families had higher prevalence of obesity compared with children from higher-income families. This inverse association was observed among the minority groups in America, such as the Indian, Pacific Island and African children. Grjibovski et al.^3^ found that maternal education and smoking were significant predictors of breastfeeding duration in the 1980s and that the association with measures of adiposity were observed in 15-year-old children and between the children with shortest and longest breastfeeding duration. Musingo and Wang^10^ did a study in which they analysed the eating habits of college students in the United States according to socio-demographic characteristics. The result showed that there were some significant differences in cooking habits, eating fruits and vegetables, residential status of students and socio-demographic characteristics. Furthermore, in terms of drinking habits, analysis by gender indicated that there were significant differences between male and female gender. In another study in the United States,^6^ it was concluded that individual socio-demographic characteristics and school-level socio-demographic composition were associated with obesity among New York City public school children.

According to Oyeyemi et al.,^11^ African specific studies on the factors associated with the growth pattern of children are needed to guide evidence and develop effective population-based interventions that can be tailored to the unique African context. Understanding these factors can be critical for establishing a road map to monitor the trend in the growth pattern of school children in African countries, especially in countries like Nigeria where school children are vulnerable to the occurrence of stunted growth or undernutrition because of severe food shortage resulting from inflation and currently because of government’s inability to pay workers’ salary and emoluments. Furthermore, today’s children are tomorrow’s world leaders and that is why nutritional assessment in the community is important for accurate planning and implementation of intervention programmes to reduce undernutrition-associated morbidity and mortality among children, including school children.^12^

## Methodology

### Design, study population and sampling

The study was conducted in Abakaliki, the capital of Ebonyi State, which is situated in the south-eastern part of Nigeria. Unemployment is a major problem in the community, especially among the youth. To make ends meet, the majority of the young men use motorcycles (called Okada) to operate public transport business. However, traditionally, the people of Ebonyi State are predominantly farmers while some do fishing as an occupation. The majority of the inhabitants of Abakaliki are characterised by low socio-economic status (SES) and live within low SES areas.

The study included only schools located in Abakaliki local government areas. The design of the study was quantitative, cross-sectional and analytical. After obtaining permission from the Ministry of Education and the principals of the schools involved, using clustering and stratified sampling techniques, one of the researchers and 10 trained research assistants enrolled 1000 learners comprising 500 each from primary and secondary schools situated in Abakaliki. The schools randomly selected through clustering included four private and four public schools. Gender selection was by stratification. After enrolment, participants were briefed about the aim of the study and the process it would involve and informed consent was obtained from their parents or guardian. Height and weight were measured using standardised procedures. For each participant, after taking the measurements, a copy of the questionnaire was completed by a trained research assistant. The questionnaire was based on the study’s objectives and validated through literature reconnaissance and several discussions and consultations among the team of researchers. The questionnaire was also pre-tested using 20 learners drawn from the randomly selected schools and amended based on the outcome of the pre-test.

### Method of data collection

Each participant was interviewed individually by 2 members of the research team and 10 trained research assistants. Measurement of weight and height was done by four nurses trained on how to conduct anthropometric measurements on children. Using the Mettler weighing scale (MWS) with a height bar, measurements were taken with the child standing erect and without shoes. Thereafter, the readings were recorded in the space provided at the bottom of the questionnaire. Although the MWS was pre-calibrated, because of the frequent movement of the MWS, it was recalibrated each time it was moved to a different location for data collection. A 10-kg dumbbell was placed on the scale and the reading was taken, the difference observed was used to adjust for the weight of participants.

The BMI for each learner was calculated using the formula BMI = mass in kg divided by height in metres (mass kg/height m). The resulting BMI for each participant and their respective ages were then measured against the BMI-for-Age Growth Charts developed by the Centers for Disease Control.^13^ The cut-off is as follows: BMI equal to or greater than the 95th percentile is obesity, BMI less that 95th percentile but greater than 85th percentile is overweight and BMI within the 5th and the 85th percentile is normal while BMI below the 5th percentile is underweight.

### Method of data analysis

Considering the nature of the questionnaire which consisted of categorical variables (except the response variable which is derived from two continuous variables [weight and height]), generalised linear model technique was used to test the association between the participants’ socio-demographic characteristics and their BMI. Goodness-of-fit test was also used to measure how well the statistical model fits into the set of observations. The measure was done using maximum likelihood estimates which were obtained by finding values that maximised the log-likelihood. The Akaike’s Information Criterion (AIC) and deviance statistic were employed as a criterion for judging the quality of the model.

### Ethical considerations

An application for permission to conduct the study was submitted to the Permanent Secretary of the Ministry of Education, Ebonyi State. Upon written approval of the application, approval was obtained from the principal or management of each of the schools involved in the study. With the support of the principals and the school teachers, each child who was recruited to participate in the study was given a consent form for the attention of and action by their parents or guardians. Only those children who returned the signed consent form from their parents or guardians participated in the study.

## Results

A total of 1000 children participated in the study and the majority of them (82.2%) were between the age of 10 and 19 years. About 32% of the children’s fathers and 39% of their mothers were either non-literate or had primary education. Only 25% and 21.4% of their fathers and mothers, respectively, were employed either in government or private establishments, while 10% of their mothers were housewives.

Regarding residence, only about 29% lived in owned accommodation and 25.8% in rented single room houses. About 37% used firewood as the mode of cooking and 43.7% used kerosene. Fifty-nine percent of the pupils has family own transport, about 76% went to school on foot and 17.7% use public transport. Over 25% lived with relatives and over 90% lived with three or more siblings. About 25.5% of the children were first born while 17.6% were last born. The minimum value of BMI was 10.82, the maximum was 31.91 and the mean was 18.43 with a standard deviation of 2.61.

As shown in Table 1, among children less than 10 years of age, 16 (10.7%) were underweight with a BMI below the 5th percentile, 13 (8.7%) were overweight while 9 (6%) were obese. Among those between 10 and 14 years, 20 (4.5%) were underweight, 24 (5.4%) were overweight while 2 (0.5%) were obese. Furthermore, among those between 15 and 19 years, 46 (12.1%) were underweight, 7 (1.8%) were overweight and 2 (0.5%) were obese. Considering participants who were older than 20 years, the majority (67.9%) had normal BMI while 9 (32.1%) were underweight.

**TABLE 1:** Body mass index-for-age percentiles.

Age (years)	Percentile (BMI norm)				Total
	< 5th (underweight)	5th to 85th (normal weight)	> 85th to < 95th (overweight)	> 95th (obese)	
< 10	16	112	13	9	150
> 20	9	19	0	0	28
10–14	20	397	24	2	443
15–19	46	324	7	2	379

**Total**	**91**	**852**	**44**	**13**	**1000**

BMI, body mass index.

Table 2 shows that gender, age and participant’s level of education are strong predictors of BMI. BMI is positively related to female gender (*p* = 0.000; beta = 0.7770) but negatively related to age and level of education. This interprets that being of female gender predicts a higher BMI, and the level of BMI increases as age and the level of study decreases.

**TABLE 2:** The relationship between gender, age, level of education and body mass index.

Parameter	Coefficient	Significance	Wald Chi-square	95% confidence interval	
**Intercept**	19.7770	0.000	20194.870	19.510	20.906
**Gender**					
Female	0.7770	0.000	35.583	0.522	1.033
Male	Reference category	-	-	-	-
**Age**					
< 10	−2.8620	0.000	91.149	−3.450	−2.275
> 20	−0.8250	0.042	4.134	−1.621	−0.030
10–14	−0.1644	0.000	44.495	−2.128	−1.161
15–19	Reference category	-	-	-	-
**Education**					
Junior secondary	0.3470	0.153	2.044	−0.129	0.824
Primary	−1.2280	0.000	19.315	−1.776	−0.680
Senior secondary	Reference category	-	-	-	-

Table 3 indicates that some of the variables, for instance, the mode of cooking, who the participants live with and how they get to school, have a strong significant change (*p* = 0.000). Although to a lesser extent, some other variables also showed significant change, for instance mother’s occupation (*p* = 0.047) and family’s mode of transport (*p* = 0.021), while father’s level of education, mother’s level of education, father’s occupation and the type of residence did not have any statistical relationship with BMI. The relationships that are significant are mode of going to school, living with both parents, cooking using firewood (*p* = 0.000), family owning a car (*p* = 0.021) and mother working as petty trader (*p* = 0.047). The direction of this relationship is however negative for petty trading, both parents living with the child and mode of transport to school, but positively related with cooking with firewood and having a family car. This means that those whose mothers work as petty traders and those who live with both parents are less likely to have high BMI. Those who cook with firewood and those who own a car are more likely to have higher BMI. Table 4 shows that having siblings and the rank of birth have no relationship with BMI changes.

**TABLE 3:** The relationship between family socio-economic profile and body mass index.

Parameter	Coefficient	Significance	95% Wald confidence interval	
			Lower	Upper
**Intercept**	19.330	0.000	18.448	20.216
**Father education**				
Non-literate	0.646	0.095	−0.113	1.404
Primary	0.281	0.316	−0.268	0.830
Secondary	0.124	0.601	−0.341	0.589
Tertiary	Reference category	-	-	-
**Mother education**				
Non-literate	0.001	0.998	−0.731	0.733
Primary	0.309	0.305	−0.282	0.900
Secondary	0.250	0.337	−0.260	0.760
Tertiary	Reference category	-	-	-
**Father’s occupation**				
Employed	0.022	0.940	−0.565	0.610
Petty trading	−0.189	0.490	−0.724	0.347
Unemployed	0.153	0.674	−0.560	0.866
Well established	Reference category	-	-	-
**Mother’s occupation**				
Employed	−0.105	0.772	−0.815	0.605
Unemployed	−0.428	0.292	−1.224	0.368
Petty trading	−0.640	0.047	−1.271	−0.008
Well established	Reference category	-	-	-
**Residence**				
Owned bungalow	0.249	0.280	−0.203	0.701
Rented bungalow	0.026	0.940	−0.650	0.702
Rented flat	0.073	0.731	−0.345	0.492
Rented small-room	Reference category	-	-	-
**Mode of cooking**				
Firewood	1.154	0.000	0.761	1.547
Gas	0.197	0.378	−0.242	0.637
Kerosene	Reference category	-	-	-
**Mode of transport**				
Family car	0.411	0.021	0.062	0.760
Public transport	Reference category	-	-	-
**Living with**				
Both parents	−0.676	0.000	−1.053	−0.298
Father	−0.288	0.486	−1.098	0.523
Mother	0.363	0.275	−0.288	1.014
Relatives	Reference category	-	-	-
**Transport to school**				
Family car	−1.26	0.001	−1.982	−0.544
Foot	−1.28	0.000	−1.692	−0.871
Public transport	Reference category	-	-	-

**TABLE 4:** The relationship between the number of siblings, birth position and body mass index.

Parameter	Coefficient	Significance	95% Wald confidence interval	
			Lower	Upper
**Intercept**	17.060	0.000	15.176	18.939
**Number of siblings**				
1–2	1.085	0.270	−0.842	3.012
3 or more	1.269	0.185	−0.605	3.143
No siblings	Reference category	-	-	-
**Birth position**				
First born	0.283	0.162	−0.114	0.680
Last born	0.339	0.135	−0.106	0.783
Only child	0.212	0.804	−1.459	1.883
Other positions	Reference category	-	-	-

## Discussion

The result shows that a great majority of the children were between the age of 10 and 19 years. This is not surprising considering the fact that in Nigeria, the system of education is referred to as 6, 3, 3, 4, meaning that a child will spend 6 years for primary education, 3 years for junior secondary, 3 years for senior secondary education and 4 years for the honours university degree programme. And because the minimum age for entry into primary school in Nigeria is 6 years, by the time that the child leaves secondary school, he or she should be about 18 years.

In this study, it was shown that the level of education of the children’s fathers and that of the mothers of about one-third was very low and the employment status of many of them was also poor. The other indicators of the children’s SES (parents’ employment status, residence, mode of cooking, family’s mode of transport, mode of transport to school and number of siblings) also showed that many of the study participants came from families with low SES. This might have been responsible for the high prevalence (14.8%) of low BMI-for-age seen in this study, which may suggest undernutrition. The results of other studies among school children in Nigeria, agree with this finding, which shows the occurrence of undernutrition in some Nigerian communities.^12,14,15,16^

In 2009, based on the results of their study, Amuta et al.^17^ concluded that the average school child in Markudi, Nigeria, is undernourished. Furthermore, the study by Manyike et al.^18^ on the prevalence of malnutrition among preschool children in Abakaliki, Ebonyi State, showed that the prevalence of global and severe acute malnutrition using *z*-score was 9.7% and 4.4%, respectfully, while that of stunting was 9.9% with male preponderance. In another study among school children in Nigeria,^14^ it was clear that an average school child in Ibadan, south-west Nigeria, is undernourished compared to the World Health Organization (WHO)^19^ standard and that of the National Standard for Health Statistics (USA) which is the reference data recommended by the WHO. The importance of food for human development both physically and mentally cannot be overemphasised.^14^ Furthermore, Amuta et al.^17^ thus observed that the need for more calories, protein and micronutrients like iron and vitamins for the children, especially those living in the slums cannot be overemphasised. They also emphasised that giving iron tablets or micronutrient fortification is not the answer to the problem of this group of children but what they need is more food which is of good nutritive value. Undernutrition, especially protein energy malnutrition, in young children continues to pose a serious problem in Nigeria, especially among the poorly educated families in low socio-economic brackets that have low purchasing power in the economy.^20^ According to Olanipekun et al.,^12^ children from low socio-economic backgrounds are not likely to have access to nutritious foods. They live in poor houses where unhygienic living standards, unsafe drinking water and unsanitary conditions of the immediate environment prevail. Such environmental factors predispose the children to infections, which adversely affect their growth and nutritional status.^20^

The result of this study shows that gender, age and participant’s level of education are strong determinants of BMI among the participants. Specifically, in this study, the female gender had significantly higher BMI scores than those of boys. This finding is consistent with similar studies that reported a higher mean BMI score among the female compared to the male gender. Amuta et al.^17^ attributed this gender differential to the earlier onset of pubertal growth spurt in girls than boys while Olanipekun et al.^12^ attributed it to the fact that girls during this age period are more homely, staying with their mothers in the kitchen and so get some extra portion of food even before the whole family is served. However, it was argued that boys at this age are more active than girls and tend to lose more of their body reserves at active play.^12^ Conversely, in a study in Rwanda, East Africa, generally, a greater proportion of male children were found to have higher BMI scores compared with their female counterparts.^21^

In this study, an inverse relationship was found between BMI level and age as well as the level of study of the participants. In other words, as the age and level of study increased, the BMI level decreased. This might be because of the fact that in developing countries like Nigeria, even in a situation of poverty or lack of food to eat, older children attending secondary schools are more likely to be involved in physical activities, like playing soccer or track events. They are also forced by family poverty or harsh socio-economic conditions to engage in child labour early in life, which is energy intensive.

The result of this study shows that children whose mothers work as petty traders and those who live with both parents are less likely to have high BMI. Children whose mothers are petty traders likely belong to families with low socio-economic backgrounds and hence are most likely not to have enough access to nutritious food, and this might affect their BMI.^12^ It is not surprising that in this study, some children who lived with both parents had lower BMI. More often than not, in Nigeria, most children live with both parents. However, children who live with both parents are more likely to have more siblings and therefore more mouths to feed. According to Owoaje et al.,^22^ children from homes with more than four children have a four fold higher risk of developing malnutrition compared to those from homes with four or fewer children. In other words, large family size is a risk factor for undernutrition and thus low BMI.^23,24^

## Conclusion

This study found a high level of low BMI among study participants. Secondly, the study also found that the BMI of school children in the community is influenced by their socio-demographic characteristics. Significantly, ‘gender’, ‘age’, ‘children’s level of study’, ‘maternal occupation’, ‘who the children live with’, ‘mode of cooking’ and ‘owning a car’ were important socio-demographic characteristics associated with abnormal BMI in the community. Based on these conclusions, the paper recommends that in order to develop an effective policy to address the problem of malnutrition among school children in Nigeria, the children’s socio-demographic characteristics must be taken into consideration, with particular emphasis on improving the SES of families living in the community.
